# Harvesting social media sentiment analysis to enhance stock market prediction using deep learning

**DOI:** 10.7717/peerj-cs.476

**Published:** 2021-04-13

**Authors:** Pooja Mehta, Sharnil Pandya, Ketan Kotecha

**Affiliations:** 1 Faculty of Technology & Engineering, C. U. Shah University, Wadhvan, Surendranagar, Gujarat, India; 2 Symbiosis Centre for Applied Artificial Intelligence, Symbiosis International University, Pune, Maharastra, India

**Keywords:** Stock prediction, Sentiment analysis, Machine learning, Deep learning, LSTM

## Abstract

Information gathering has become an integral part of assessing people’s behaviors and actions. The Internet is used as an online learning site for sharing and exchanging ideas. People can actively give their reviews and recommendations for variety of products and services using popular social sites and personal blogs. Social networking sites, including Twitter, Facebook, and Google+, are examples of the sites used to share opinion. The stock market (SM) is an essential area of the economy and plays a significant role in trade and industry development. Predicting SM movements is a well-known and area of interest to researchers. Social networking perfectly reflects the public’s views of current affairs. Financial news stories are thought to have an impact on the return of stock trend prices and many data mining techniques are used address fluctuations in the SM. Machine learning can provide a more accurate and robust approach to handle SM-related predictions. We sought to identify how movements in a company’s stock prices correlate with the expressed opinions (sentiments) of the public about that company. We designed and implemented a stock price prediction accuracy tool considering public sentiment apart from other parameters. The proposed algorithm considers public sentiment, opinions, news and historical stock prices to forecast future stock prices. Our experiments were performed using machine-learning and deep-learning methods including Support Vector Machine, MNB classifier, linear regression, Naïve Bayes and Long Short-Term Memory. Our results validate the success of the proposed methodology.

## Introduction

The opinions of others often play a role in an individual’s decision-making process and this was especially so prior to the advent of the World Wide Web. Recommendations from friends, colleagues and co-workers played an integral role in everyday decision-making. However, more people are using the Internet to make their views accessible to strangers. People who use a social network or social media sites like Facebook or Twitter communicate their opinions on specific topics, such as news, movies, events, or a particular product (Sun & Ng, 2014). Sentiment Analysis (SA) is a field of study in which people’s views and expressions are classified as positive, negative, or neutral. There are a number of definitions found in the literature, but SA is best defined as analysis used to extract data based on user sentiment. The analysis of sentiment and emotions, known as opinion mining, is the study of opinions, thoughts, experiences, feelings and behaviors in text form (Liu, 2012).

**Figure 1:**
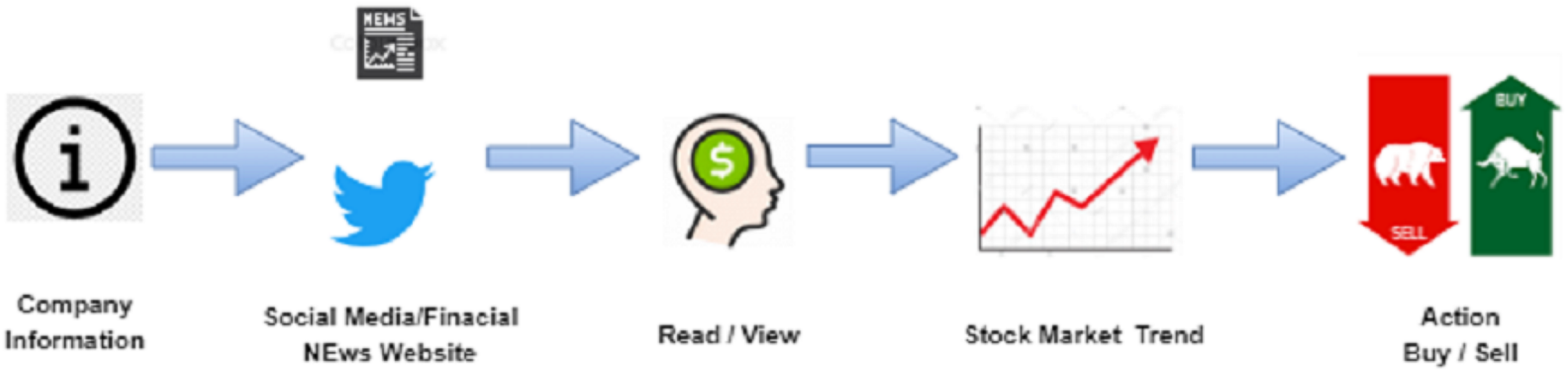
An illustration of how social media and finance news has impact on stock market movements.

An entire market has been established to detect financial market sentiments (Xing, Cambria & Welsch, 2018a). Stock prediction is the crucial for researchers and investment planners. Stock prices have always had short term and long-term fluctuations. Various machine learning techniques can provide more accurate and reliable results related to the share market price, however the development of a useful stock forecasting model remains difficult. The current stock market (SM) is affected by social mood and historical prices, which can play a significant role in the movement of stock prices within the social world. Daily news articles also play a significant role in predicting stock prices and are responsible for the distribution of information related to the company or budget to the public and indicate their SM trading strategies. The use of news articles to forecast SM movement is the focus of this research. News articles on a particular industry typically explain how the organization performs and what will happen to the shares. As more financial data becomes available, it is possible to use it in basic research to predict stock price changes at a much faster rate (Ritesh, Chethan & Jani, 2017; Pandya et al., 2018; Awais et al., 2020; Sur, Pandya & Sah, 2020; Barot, Kapadia & Pandya, 2020). Figure 1 illustrates how social networking sites and financial market news impact the listed SM data.

Changes in an equity market are sparked by human choices based on beliefs regarding future effectiveness. The share market prediction is based on interest rates, commercial sectors, and key markets that change trading demand and supply. A poor investment could easily result in considerable losses for investors, especially if they continue to make the wrong choices. For stock traders, a useful predictive model is of significant importance. Investors desire techniques that can make efficient use of massive amounts of data. Data for the pattern analysis includes a mixture of features, such as textual data and SM data, where some features are more meaningful while others are as not predictive.

Our research aims to use social media knowledge to establish a framework for analyzing and predicting historical trends in stock prices. We explain how financial news is related to and used with stock price prediction, providing a useful market movement study. One widely used technique is to compare the SA of the articles and their correlation to behavior in stock price. Here, we try to answer whether investor sentiment can help predict changes in stock prices.

**Figure 2:**
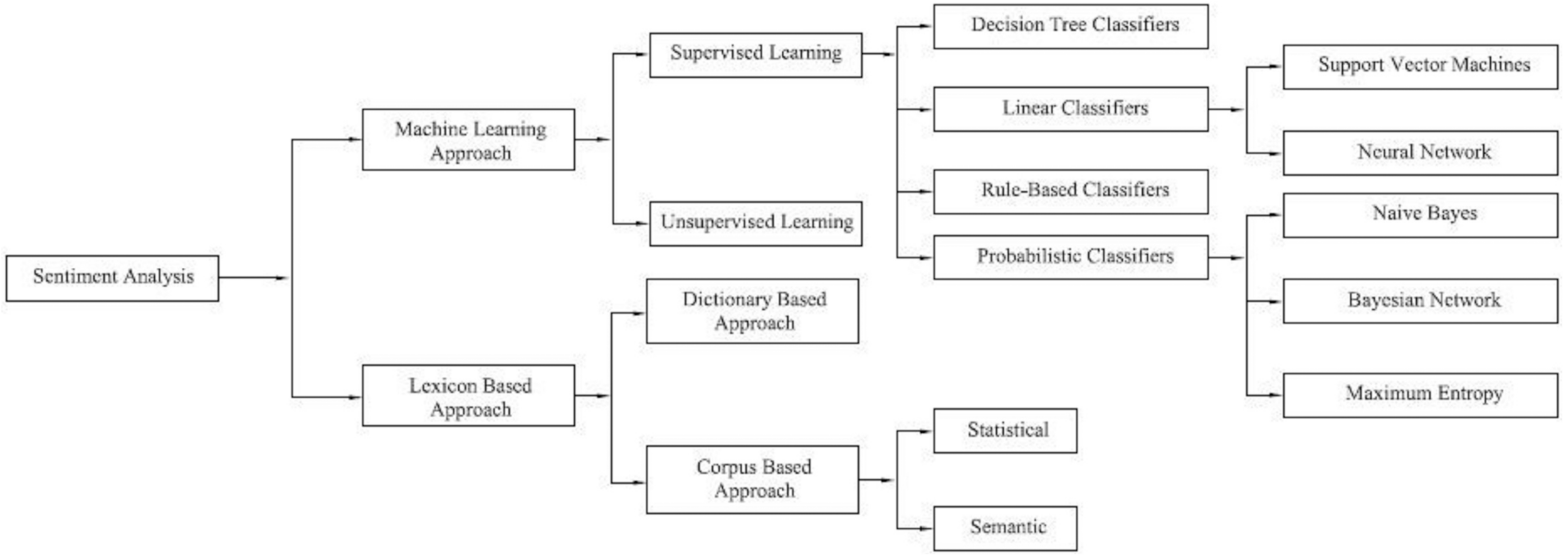
Classification techniques of sentiment analysis.

Stock investors and financial planners use various time series methods for market prediction techniques. There is a higher possibility that a stock price will continue to rise if the sentiment is positive, and the stock price will fall if it is malicious. Figure 2 shows the classification techniques of SA.

The rest of the article is organized as follows: “Related Work” discusses past research on this topic. “Methodology” describes data preprocessing, the proposed model of the proposed system, and the Long Short-Term Memory (LSTM) framework. “Experiment Setup and Results” describes an experiment involving SA and its ability to predict the SM with LSTM. This section includes output result details and different machine learning techniques to predict SA results. The concluding remarks and the possibility of future enhancements are discussed in “Conclusions and Future Scope”.

## Related work

Opinion mining or sentiment is based on the people’s specific views. SA plays a role in extracting information for many applications, such as product reviews, health care, politics, and surveillance. Predicting stock movements is an important and active field of study and involves accurate predictions. There has been considerable progress in recent years to develop predictive models for the global SM. Many standard approaches and methods are used in literature reviews, including time series prediction analysis. Many machine learning modeling techniques are used as well. We gathered some of the information about SM analysis strategies from the literature. Milosevic (2016) and Pandya, Sur & Kotecha (2020) proposed the machine learning technique to forecast long-term investments. This technique results in an *F*-score of 75.1% for all 28 selected financial variables. Feature selection allows the number of features to be decreased to 11, while performance can be improved to an *F*-score of 76.5%. Alexander, Ilya & Alexey (2013), proposed the SVM and Neural Network (N.N.) algorithm to predict the SM. This application chooses DJIA and S&P500 indicators in its predictions using a lexicon-based approach for analysing psychological states. The Twitter data set and DJIA stock data were applied and successfully deterimined the success of the stock. The best average accuracy can be achieved by using the Support Vector Machine (SVM) algorithm to predict the DJIA indicator by 64.10%. Lai & Liu (2014) discussed the model in stock forecasting using SVM and the Least Square SVM.

Mihir, Sumedh & Anmol (2020) and Mehta & Pandya (2020) conducted a study regarding the relationship between public sentiment and stocks using an analysis of sentiments and a machine learning approach. The three different prediction techniques, General Autoregressive Conditional Heteroskedasticity (GARCH), Support Vector Regression (SVR) and Least Square Support Vector Machine (LSSVM) were combined with the wavelet kernel to form three new GARCH (WL GARCH) based Wavelet algorithms, Wavelet-based SVR (WL SVR) and Wavelet-based Least Square Vector, Support Machine (WL LSSVM) to address the problem of the non-linear and non-parametric financial time series. Using the Hang Sang Index, Dow Jones and the Shanghai Composite Index, these wavelet-based algorithms were differentiated. The best results were obtained without the LS-SVM dependent Wavelet kernel. Xing, Cambria & Welsch (2018b) and Sur, Pandya & Sah (2020) present the scope of Natural language based financial forecasting (NLFF) by using Natural Language processing or programmatic study. NLP techniques are quickly growing, progressively establishing an area of research dealing with of financial forecasting focused on NLFF or SM analysis from the application point of view.

In communication, emotion plays a significant role in communicating effectively. Jasmina et al. (2013) and Pandya, Sur & Kotecha (2020) studied whether Twitter feeds are a useful source of data for predicting trends in stock closing prices, sharing public sentiment about companies, and their products. This paper proposed the sentiment probability for positive and negative expressions or opinions to be measured for forecasting SA in finance. By using the Granger causality test, a few days’ data can indicate trends in stock price. Adopting the SVM also defines tweets as positives, negative, or neutral sentiments, resulting in increased predictive ability on the SM. Many efforts have been made (Sushree et al., 2018; Ghayvat et al., 2019; Pandya & Ghayvat, 2021; Ghayvat, Pandya & Awais, 2021; Srivastava et al., 2021) for SM prediction; predicting the firm’s effective SM price with Twitter data streaming shows the collection in actual data time. The research reveals how SA of public mood extracted from Twitter or any social media feed can effectively predict movements in independent share prices. A stream-based technique has been adapted to use the Incremental Active Learning Method, which allows the algorithm to pick new training data. Moreover, this analysis's economic impact also used Recurrent Neural Networks (RNNs) LSTM experiment.

Analyzing sentiment helps to evaluate the emotion’s impact in a textual context for positive decision analysis. Bhuriya et al. (2017) developed models of regression to forecast TCS market price based on five attributes, i.e., open, large, small, close price and volume. The research evaluated the linear, polynomial, and radial base function impact for regression models based on the expected outcomes' confidence values. The linear regression method overtook other techniques and obtained a confidence score of 0.97. With the growing world market economy, stock prices fluctuate very unexpectedly. Even with experience, stock prices are difficult to forecast due to historical patterns and previous stock prices. Mate et al. (2019) presented the stock price prediction by news SA, which predicts stock price changes. They also introduced the idea of using SA to rate articles with single combined strings and string a positive, negative, and neutral rating. The performance of the SA is fed to stock-market prediction to any machine learning models. Patel et al. (2015, 2020) discusses the application of forecasting stock and share price index movements in an Indian equity market using a machine learning framework. They used four forecasting models to analyze the data: (1) ANN, (2) SVM, (3) Random Forest and (4) Naïve Bayes. Each has two input methods. The application analyzes the accuracy of each of the two input techniques from each predictive model. Reviews of two organization’s stocks, namely Reliance Industries and Infosys Ltd., and two stock price indices CNX Nifty and S&P Bombay Stock Exchange (BSE) are taken from 2003 to 2012. Khedr, Salama & Yaseen (2017) presented analysis and an optimized framework designed to focus on the minimum error ratio and improve prediction accuracy while forecasting share price performance patterns. Methods including financial news SA and historical SM values have been used to form the prediction model. The methods mentioned above have effectively used various forms of market and company data and have provided the best results compared to other studies conducted in the past. They used the data set of three companies with stock values. First, they studied the news sentiment to use naïve Bayes classifier to have the text polarity, which provided a predictive accuracy between 72.73% and 86.21%. Second, they combined past stock prices and news polarities to forecast future stock prices, resulting in an almost 89.80% predictive accuracy. Chen et al. (2018) compared standard N.N. price prediction with deep learning using CSI 300 share value data from the Chinese SM and observed that deep learning prediction performance was higher than the conventional N.Ns.

Carosiaa, Coelho & Silva (2020) addressed SM movements based on news and foreign events that strongly affected the market value of certain companies. The research focused on an analysis of the Brazilian SM action on Twitter by SA considering three perspectives: (i) An absolute number of Tweet emotions; (ii) Tweet feelings weighted by favorites; and (iii) Re-Tweet weighted feelings. They used the Multilayer Perceptron method for their experiment to achieve SA in Portuguese. Deep learning algorithms also play a vital role in the field of SA. Santos & Gatti (2014) established a deeply convolutionary N.N. based on the Stanford Sentiment Treebank of film review sentences as well as the Stanford Twitter Sentiment Corpus with Twitter messages. They achieved an accuracy of over 85% in both corpus sentence sentiment forecasting. Financial data changes are based on a multitude of correlated variables that are continually changing. Yoshihara et al. (2014) discussed an SA technique using deep learning to forecast the financial market by combining RNN with Deep Belief Networks (DBN). The expected financial market behavior predicted by SA showed that the error rate decreased from 47.30% to 40.05% on average, which compares favorably to SVM and DBN strategies. Jiang (2020) presented an analysis of deep learning models for forecasting the SM. He categorized different frameworks of the N.N., common evaluation metrics, and their integration and reliability. This study represented the latest development in SM prediction using various approaches. Pang et al. (2018) and Sun (2016) proposed that N.N. methods be developed to achieve higher stock data predictions and that a deep learning model of LSTM’s N.N. should be used to predict financial markets. Their accuracy for the Shanghai A-shares composite index was 57.2% and 56.9%, respectively. Khan et al. (2020) implemented data algorithms using social networks and business news to evaluate the effects of this data on the accuracy of SM forecasts over 10 days. The selection of features on the data sets and the elimination of spam tweets were conducted to improve efficiency and predictive accuracy. Finally, deep learning was used to achieve greater predictive accuracy and some classifiers were installed. The experimental results indicated that using social networking and economic news resulted in 80.53% and 75.16%, predicted accuracies, respectively. This study suggest that SMs are difficult to evaluate in New York and Red Hat, while stocks in New York and IBM are highly affected by social media; shares in London and Microsoft are more highly affected by financial news. The random forest classifier was found to be accurate and its ensemble achieved the best accuracy at 83.22%.

Yu & Yan (2020) presented data on financial commodity prices as a one-dimensional series created by the projection of a natural system consisting of multiple indicators into the time dimension and reconstruction of stock prices using the time series phase-space reconstruction (PSR) technique. A predictive model based on DNN was developed using the PSR system and an LSTMs was developed based on deep learning to forecast market prices. Deep learning methods are an extension of the ANNs, that have experienced sudden growth to forecast financial time series.

## Methodology

Our analysis was intended to forecast the Indian SM from 1 October 2014 to 31 December 2018 via the different SA of various social media sources including Moneycontrol, IIFL, Economic Times, Business Standard, Reuters, and LiveMint. The following steps were taken: (1) data collection; (2) data pre-processing; (3) SA of news; (4) application of the various machine learning approaches to compare the data; (5) SM forecasting using the Deep Learning Model.

### Data collection

**Figure 3:**
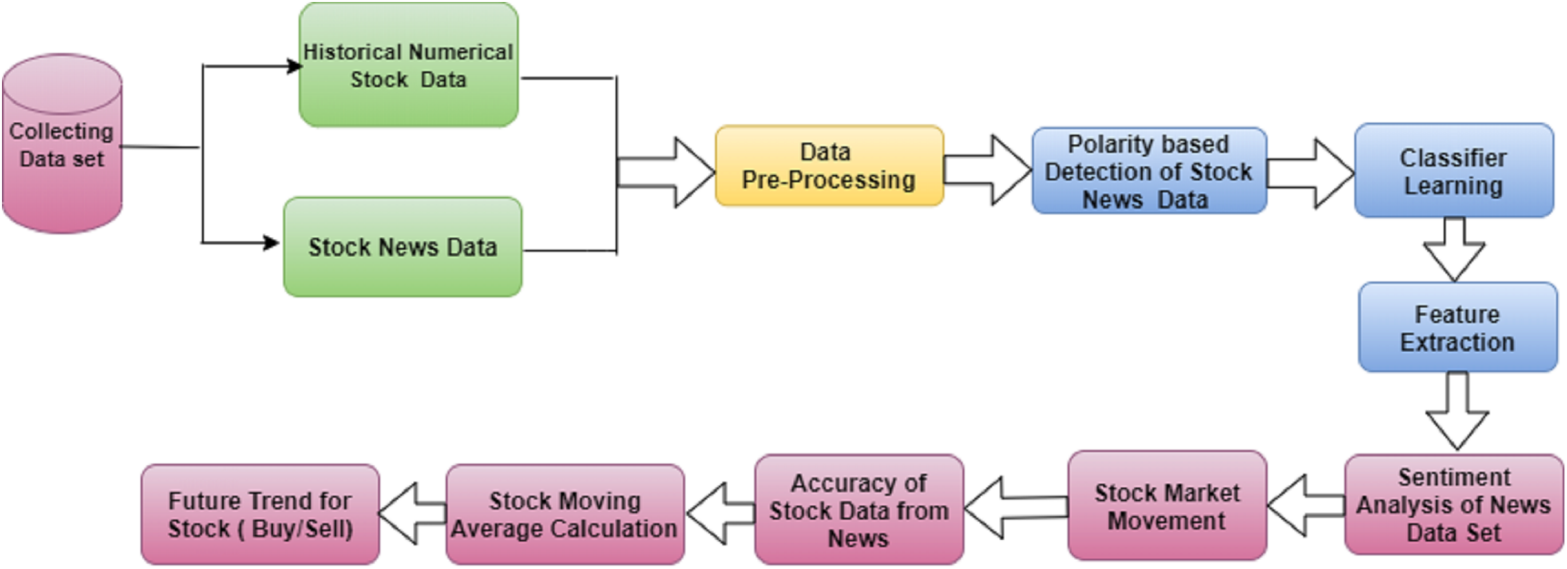
The proposed model to predict stock data using sentiment.

We used two data sets collected from different data sources. The news from Moneycontrol, IIFL, Economic Times, and Twitter was taken as one set. NSE Stock data for each stock was considered to be raw data and included the following parameters: (i) date; (ii) time; (iii) stock open value; (iv) stock high value; (v) stock low value; (vi) comparable stock value; and (viii) stock volume traded at a given interval. This system design for news articles was proposed for generating market trends from stocks. Figure 3 describes the proposed model for SM predictions using SA. Figure:- pre-processing, sentiment classification, and the LSTM method were the main modifiers used to predict stock price.

### Data pre-processing

The first step in pre-processing was to synchronize the text by transforming it into lowercase characters. There may be various causes in the text, and this can impact classification. It prepares input data to be processed by the classifier of the sentiment. This process consists of data cleaning (i.e., link removal, special symbols, emoticons, etc.), stop word elimination, parts of speech, stemming, and tokenization. The output from the pre-processor subsystem was used by the sentiment classifier method, which uses a machine learning approach (Naive Bayes, ME, SVM, or MLP) to recognize the emotions of each document individually. News & tweets include views about the data shared by various users in different ways. The research survey dataset used here was classified into two major groups. Negative and positive polarity makes it easy to identify the influence of different characteristics.

We then evaluated the polarity between −1 and 1 of the data, which was further defined as:

Polarity = 0: Neutral Sentiment

Polarity < 0: Negative Sentiment

Polarity > 0: Positive Sentiment

Then, the SA module calculated the sentiment of given news related to stock data. This module used three perspectives to consider the predominant sentiment as shown in Table 1.

**Table 1:** Predominant sentiment formulas.

Precision	Recall	*F*-score	Accuracy
∑Ŧp ∑Ŧp +∑Fp	∑Ŧp ∑Tp + ∑Fn	2∑ Ŧp/(2∑ Ŧp + ∑FP + ∑FN)	∑ Ŧp + ∑Tn ∑Ŧp + ∑Tn + ∑Fp + ∑Fn

The overall result of each of these four significant observations is a value between −1 and 1. Values between −1 and 0 are negative, and 0 to 1 indicate that the principal opinion or sentiment is positive. During each trading day, stocks provided some essential trade details, including available price, close price, revised close price, highest price, lowest price, and volume. The calculated close price normally reflected the stock price of the trading day and others with several adjusted close costs over the trading period. We define as:

s1, s2, s3,…,sT

sT is the close stock price on *t* trading day, which are overall trading days. The stock price on a certain trading day will go up or fall relative to the previous trading day. Here, we used the closing price change from the last two sequential trading days for analysis. The trading condition was classified as:

(1) $$Ft=\left\{ \begin{array}{cc} 1 & ifSt>St-1\\ 0 & ifSt<St-1 \end{array}\right.$$

If Ft is 1, it indicates that the price may go up on the next day; a value of 0 it indicates that the price may fall or continue along the same trajectory.

Next, the appropriate machine learning techniques were decided for analyzing sentiment classifications on the SM data. The results may be positive and negative.

The sentiment expectation is combined with the SM data used for machine learning training algorithms, including Naive Bayes, Maximum Entropy, linear regression, support vector, and deep learning.

For the deep learning model, a very well-labeled training data set in Comma-Separated Value (CSV) format is necessary. The labeled dataset was first assembled (text with a sentiment label), and the model was trained using that dataset; then, after training, the text was input into the system resulting in a text sentiment value.

**Deep Learning Model:** Our study's objective was to build a reliable predictive model for stock movement.

**Long Short-Term Memory (LSTM):** These networks are a form of RNN likely to learn sequence prediction dependence. It is a unique form of RNN able to learn long-term dependencies. Throughout this segment, we explored the LSTM network classification.

**Figure 4:**
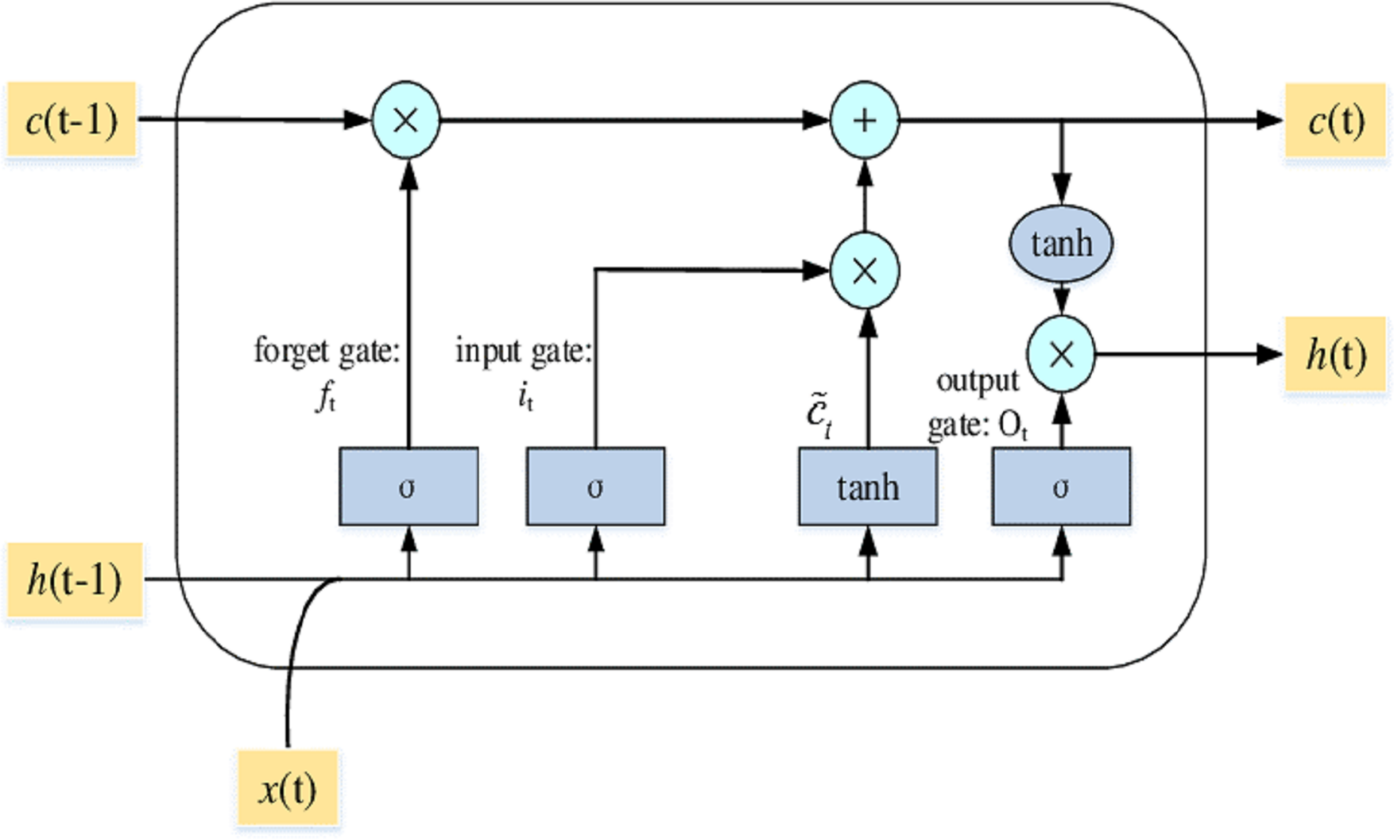
The LSTM model with its three layers.

LSTM’s allow RNN to keep track of their input data over a long time. LSTMs place the relevant data in memory similar to a computer because the LSTM can read, write, and eliminate information from its memory. There are three layers in an LSTM: the input, forget, and output gates. All gates specify whether to include new input (input gate) or not, to exclude the information because it is not necessary (forget gate), or to allow it to affect the output at the current time stage (output gate). Figure 4 shows an illustration of an LSTM RNN with its three gates.

LSTM’s have a chain structure and run through N.N. gates and layers like other RNN methods. The layout of the LSTM is built in a way that runs through the entire LSTM cell state; the value is also modified by gates that have worked by either enabling or suspending the application of data to the cell state.

A memory cell contains the input gate (it), output gate (ÖT), and forget gate (ft). The gates are used to manage the connection of memory cells to itself and neighboring cells. At time t, the input vector is xt, the output vector is ht, and the memory cell's vector historical knowledge memorizing is Ct. The input is xt at time t, the output is ht, and historical information that memory cell memorizes is Ct. Weight is denoted by W and b are biased. The input layer manages the information at time t; the forget gate will choose time t to recognize or forget historical information. The output layer controls what more information will also be passed to the next layer.

The memory cell block framework is represented in Fig. 4. Considering the sequential feature of the memory cell, Ct includes all input information from time 0 to time t that reflects the condition at time t.

(2) FT=σ(Wf∗[ht−1,xt]+Bf)

In the above Eq. (2), the forget gate (ft) is defined by input xt and output ft−1 of the last time. The value of the forget gate runs between 0 and 1. When ft = 0, the previous value is forgotten in the calculation. ft = 1, the forget gate keeps previous information.

(3) ΔCt=tanh(WC⋅[ht−1,xt]+bC)

From Eq. (3), ∆Ct is between −1 to 1 using input text and ht−1. Equation (4) implied that the input layer controls xt and ht−1, similar to the forget layer. When it = 0, ∆Ct can be excluded, and it = 1, then ∆Ct will be considered Ct.

(4) it=σ(Wi⋅[ht−1,xt]+bi)

In Eq. (5), two components represent the state vector Ct. The first element is the state vector of the previous time node Ct−1, operated by the forget gate. And the second part is ∆Ct, which depends on how much input the gate is allowed.

(5) Ct=ft∗Ct−1+it∗ΔCt

ÖT = output gate is equivalent to the input layer and forget layer in Eq. (6). The size of the output ÖT specifies what more possibility other layers will produce and receive the state vector Ct.

(6) $$\ddot{O}T=\Sigma (Wo\cdot [ht-1,\;xt]+Bo)$$

The ht output vector is the combination of the ÖT output gate and the Ct cell state. Ct has to be converted by the tanh function, and then the output gate has to decide the output portion in Eq. (7).

(7) $$ht=\ddot{O}T*tanh(Ct)$$

LSTM networks involve memory cells that can handle a condition over time using memories and chain systems that manage the information flow into and out of the brain. The model input is the closing value of the previous day, and the target value was set to the opening value of the current day.

We are predicting the future closing price of different companies with the help of LSTM. This prediction will be based on previous data and day-by-day forecasts of the future. The system input is the closing value of the previous day, and the target value was set to the opening value of the current day.

### Stock moving average method for prediction

The stock moving average method is a technical research tool wherein the actual index data is compared to its average measured throughout a period. The standard period for stock forecasting is 5-days, 10-days, 15-days, 21-days, 50-days, and so forth. The key benefit of the moving average stock predictor is that it provides a perfect line and manages to reduce the amount of distortion, mostly on price movement instead of other predictor types. The Sensex is established to display the sentiments of the entire economy. These are significant, well-established, and financially sound organizations from primary industries. The moving average is evaluated using the stock price information to extract whether the Sensex will rise or fall on the following day. By adding the closing price and then dividing this sum by the number of time intervals, the stock's moving average is measured in Eq. (8).

(8) $$Pt=\frac{St-1+st-2+St-3+\cdots +St-n}{N}$$

Pt = Prediction for the following year

St−1 = Specific occurrence for periods up to 'n' in the past period,

N = Average number of periods.

The proposed predictive method included the 5-day, 10-day, and 15-day, and 1-month, 3-months, and 6-months moving average prediction for BSE Sensex and Infosys company for 2014–2020.

## Experiment setup and results

Our research findings provided some critical perspectives. Our objective was to evaluate the accuracy of LSTMs in forecasting the stock industry. The conceptual system for the proposed method of machine learning and deep learning for stock forecasting with performance analysis is explained here and refers to a highly accurate and useful stock-market classification model. We used a vast number of different models to measure accuracy, but most importantly, we focused on the deep learning technique of LSTM. This section described this paper’s proposed work: sentiment polarity score, performance matrix, and LSTM with improved approaches. Specifically, we chose LSTM as our reference and for use in various experiments.

### Collection of data set

**Figure 5:**
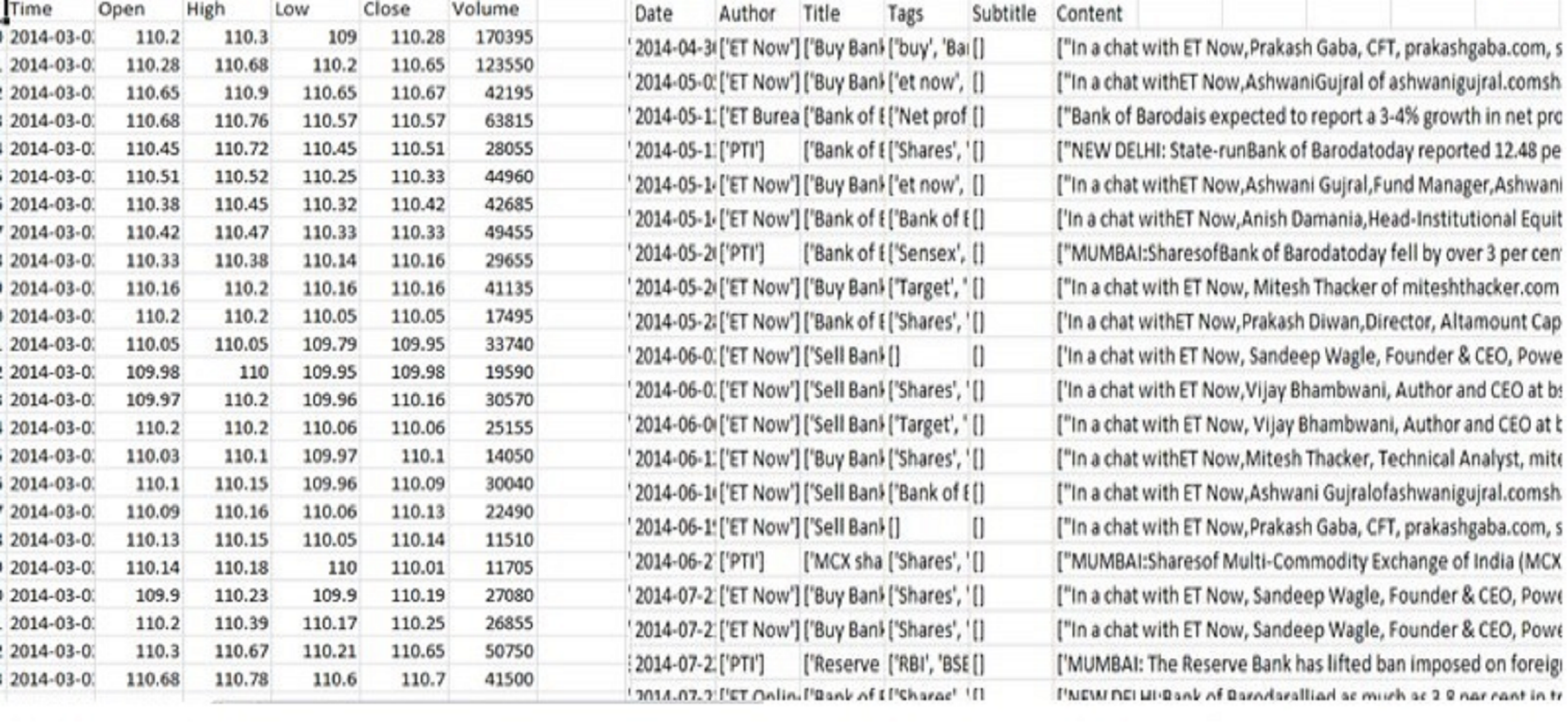
Stock and news dataset.

We combined the Indian stock data over the past 6 years (Fig. 5) and included the news data set with attributes including date, author, title, and content. The stock data set contained attributes including open, close, low, high, and volume. The collected information was stored in a spreadsheet CSV file for ease of management, smaller size, ease of production, and easier enforcement.

### Input data preprocessing

**Figure 6:**
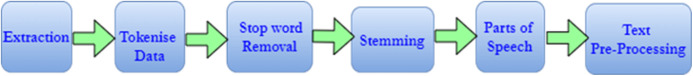
Pre-processing phase.

The data set was preprocessed. Figure 6 shows the preprocessing steps that include deletion of the stop terms/words and data collection stemming. In this step, the data cleaning was conducted, including the deletion of HTML tags (i.e., < >), specific stop words (i.e., an, the), working with punctuation marks (i.e., numerical values, spaces, comma, semicolon), and tokenizing the remaining terms in the data sets.

The presented framework used historical open and closed stock prices. The sentiment polarity representation has been used to represent these prices in terms of positive and negative. According to the research carried out at Stanford University (Yu & Yan, 2020), the following sentiment polarity values have been considered in the conducted experiments.

$$\text{Sentiment Polarity}=\left\{ \begin{array}{l} \text{0 (more negative)}\\ \text{1 (Negative)}\\ \text{2 (Neutral)}\\ \text{3 (Positive)}\\ \text{4 (more positive)} \end{array}\right.$$

**Figure 7:**
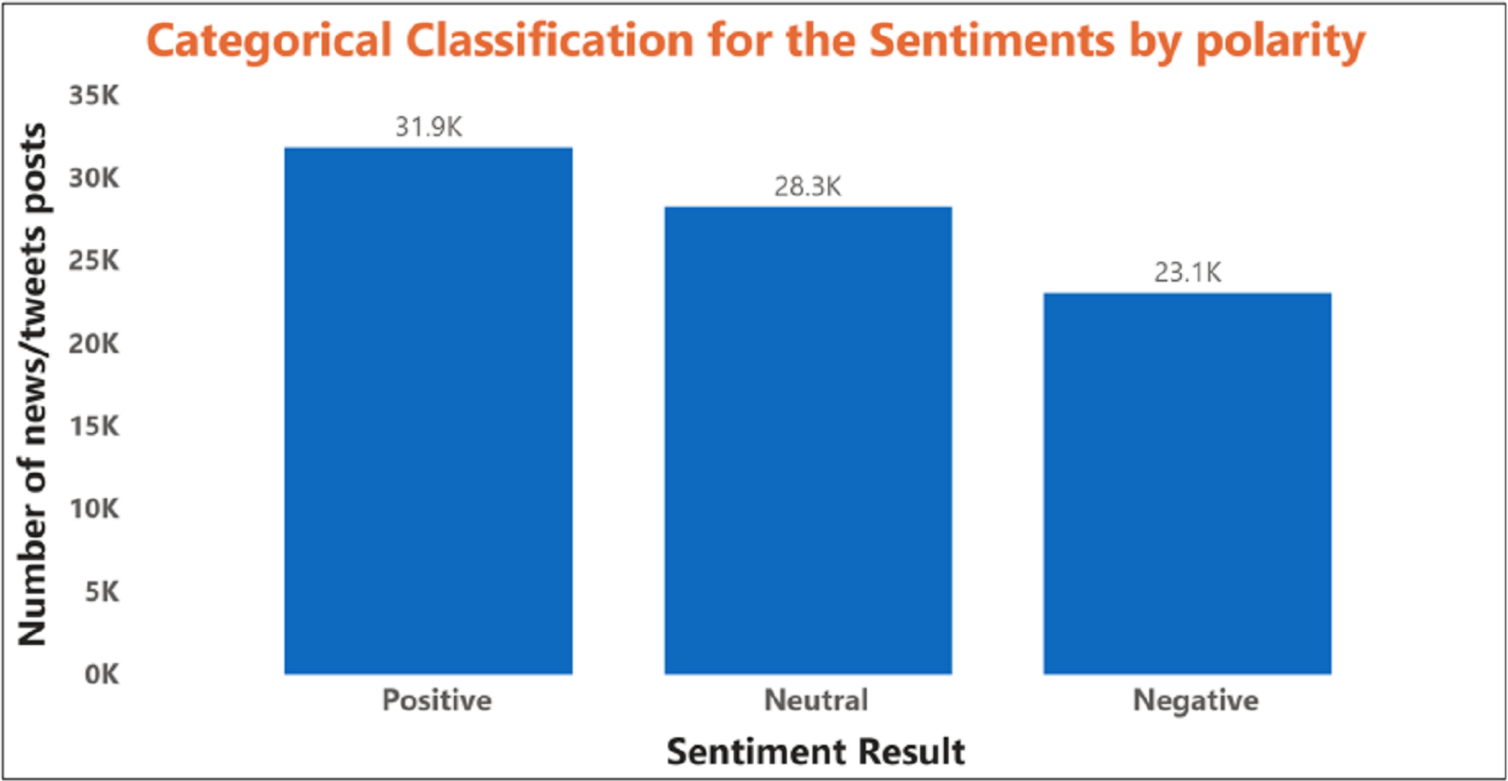
The illustration of the polarity based sentiment classification with several stock news data.

The behavior obtained is based on the statistical parameter. This model can still be improved using specific attributes that can directly affect stock prices and data mining prediction algorithms. Figure 7 shows the polarity of the sentiment of the SM data.

The relationship between the expected news sentiment and the stock data set was examined at this stage. The closed price of daily stocks was adjusted accordingly. If the news sentiment was positive, there was a greater chance that the stock price would incline continuously; otherwise, it may fall. The count of positive, negative, and neutral polarity represented the words and was proposed to assess the overall stock news data, showing the predicated polarities of sentiment as positive, negative, or neutral for several news data. Table 2 shows the prediction result of stock forecasting using sentiment polarity.

**Table 2:** Dataset for stock prediction based on sentiment polarity.

Date	Open	High	Low	Close	Trend	Future prediction
10/1/2017	152.25	152.25	152.2	152	Positive	Positive
09/2/2017	183.7	183.72	183.4	183.7	Positive	Negative
10/3/2017	159	159.1	158.9	159	Negative	Negative
05/4/2017	173	173.05	172.95	173.0	Positive	Positive
03/5/2017	187.35	187.35	187.3	187.35	Positive	Negative
25/5/2017	176.6	176.6	176.1	176.25	Negative	Negative
07/6/2017	175.65	175.75	175.5	175.65	Positive	Negative
23/6/2017	162.05	162.2	162.2	162.2	Negative	Positive
07/7/2017	163.65	163.7	163.6	163.7	Positive	Positive
27/7/2017	162.4	162.4	162.25	162.25	Positive	Negative
07/8/2017	160.05	160.05	159.9	160	Negative	Positive
29/8/2017	140.9	141	140.8	140.85	Negative	Negative
14/9/2017	144.1	144.9	144.1	144.85	Positive	Positive

**Sentiment Analysis Result:** The average sentiment estimates the regular sentiment of any topic over a given period. Here, the system considers using given views/opinions, the primary sentiment as accuracy, precision, F-score, and recall. The system first evaluates the performance matrix in this phase in which we find the training data set. Table 3 shows the form of a performance matrix.

**Table 3:** Performance matrix.

	True	False
True	True positive	True negative
False	False positive	False negative

For example, consider taking this Table 1; we have the following Table 4 that shows data to calculate sentiment measurements.

**Table 4:** Number of dataset to calculate performance matrix.

	True	False
**True**	34,500	4,500
**False**	17,000	24,200

The Table 5 shows the result of the performance matrix.

**Table 5:** Results of performance matrix.

Precision	Recall	F-score
88.46%	66.99%	76.24%

Stock forecasting performance using different financial news and social media sentiment data indicate that the result is more accurate and reliable than previous predictive attempts. Classification of the SA used algorithms to construct a model of classification.

**Figure 8:**
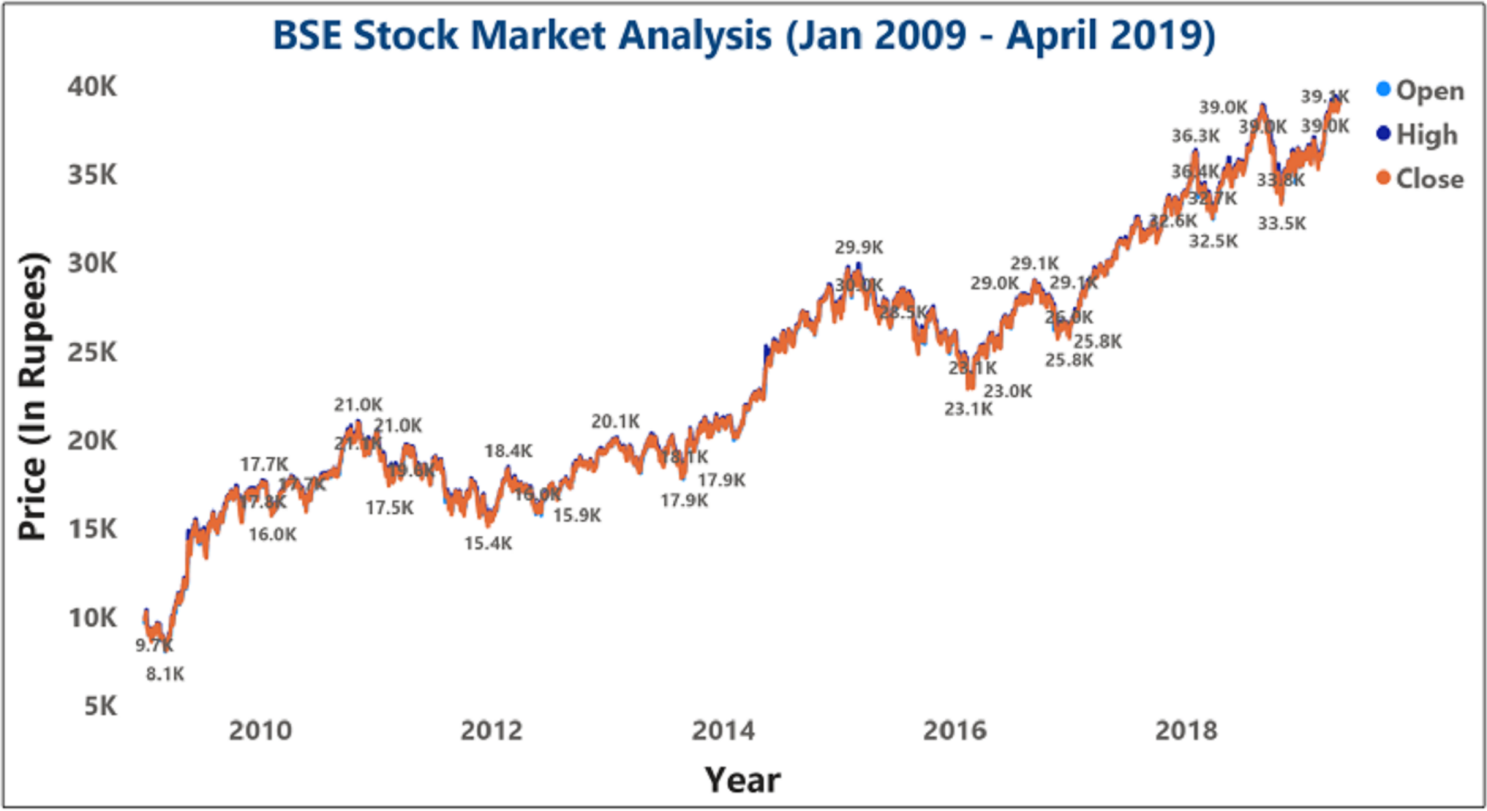
Analysis of BSE sensex opening and closing opening and closing stock price data.

The objective is to forecast a company’s stock price on analysis using previous day information and financial news, known as the Properties. As shown in Fig. 8, various classification approaches have determined the BSE SM’s stock forecasting accuracy to be between 85% and 93%. The BSE SM analysis of stock price data between the 10 years (2009–2019) for various BSE open and close prices were also evaluated.

**Figure 9:**
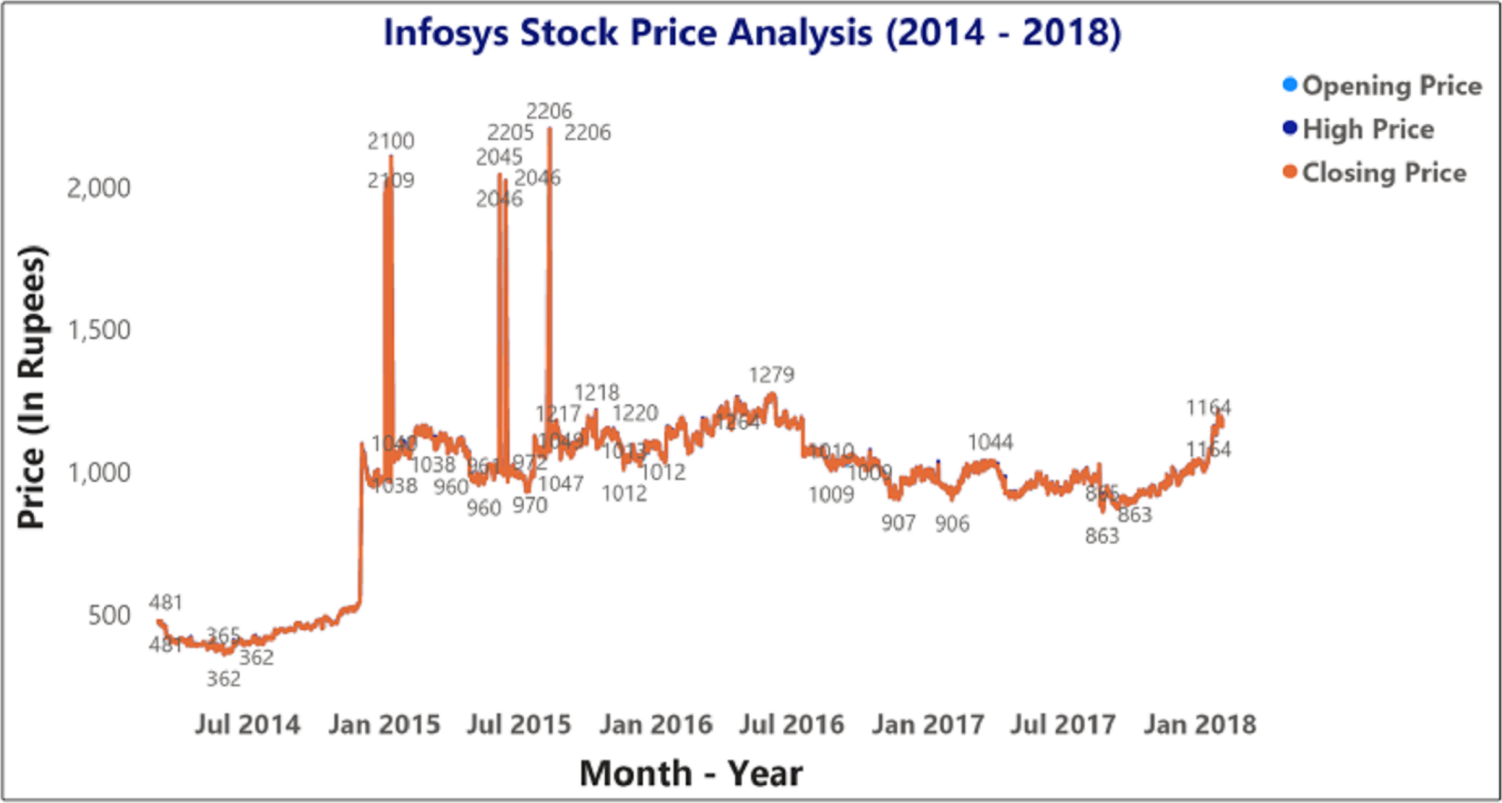
Analysis of opening and closing opening and closing stock price data of the Infosys Company (2014–2018).

**Figure 10:**
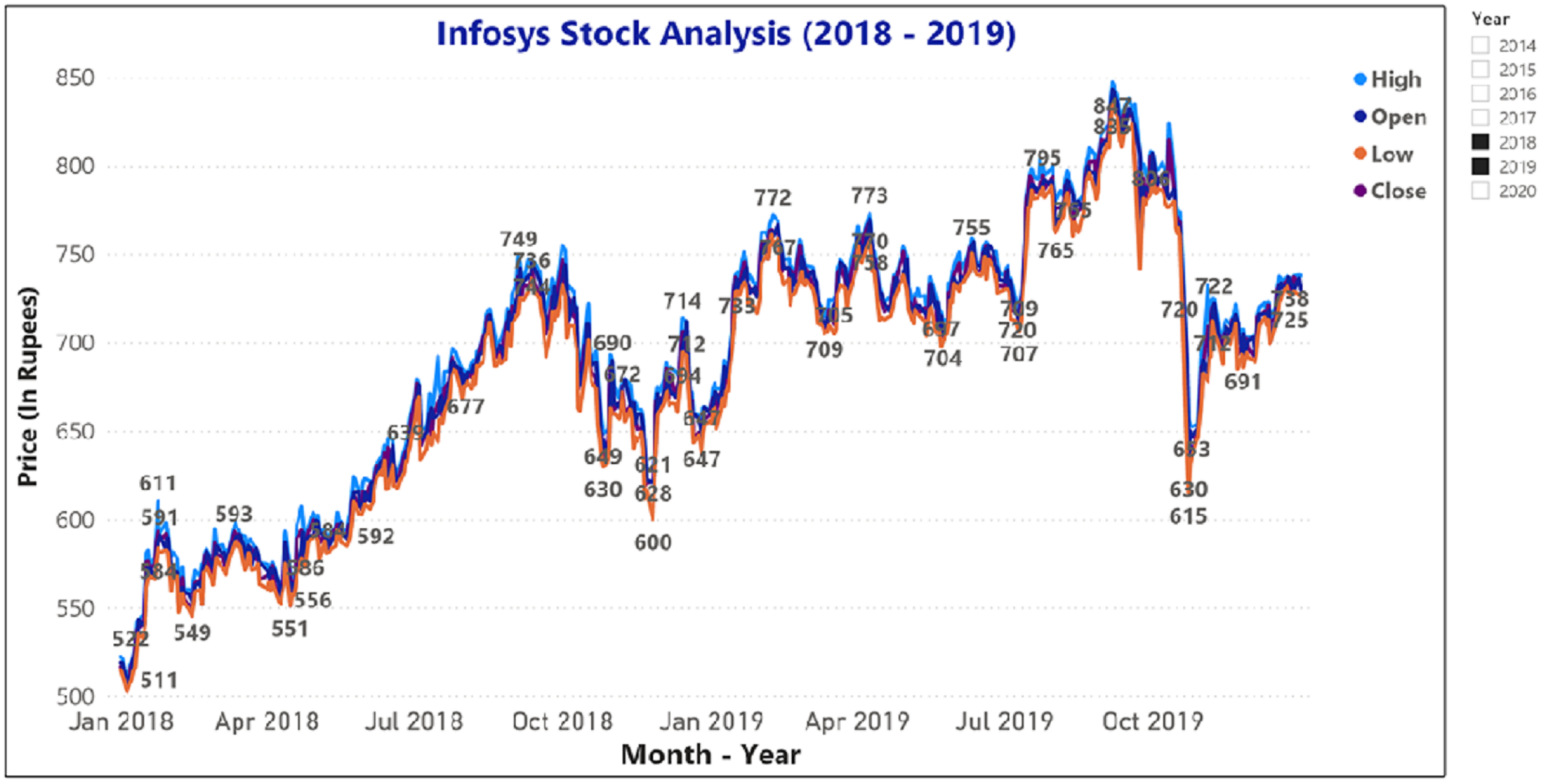
Analysis of opening and closing stock price data of Infosys Company (2018–2019).

Figures 9 and 10 represents the graphical analysis of the Infosys company’s opening, high, and closing price data for the year-span: (i) 2014–2018, (ii) 2018–19. Figures 8–10 shows that the opening and closing prices have been done concerning stock price values.

**Figure 11:**
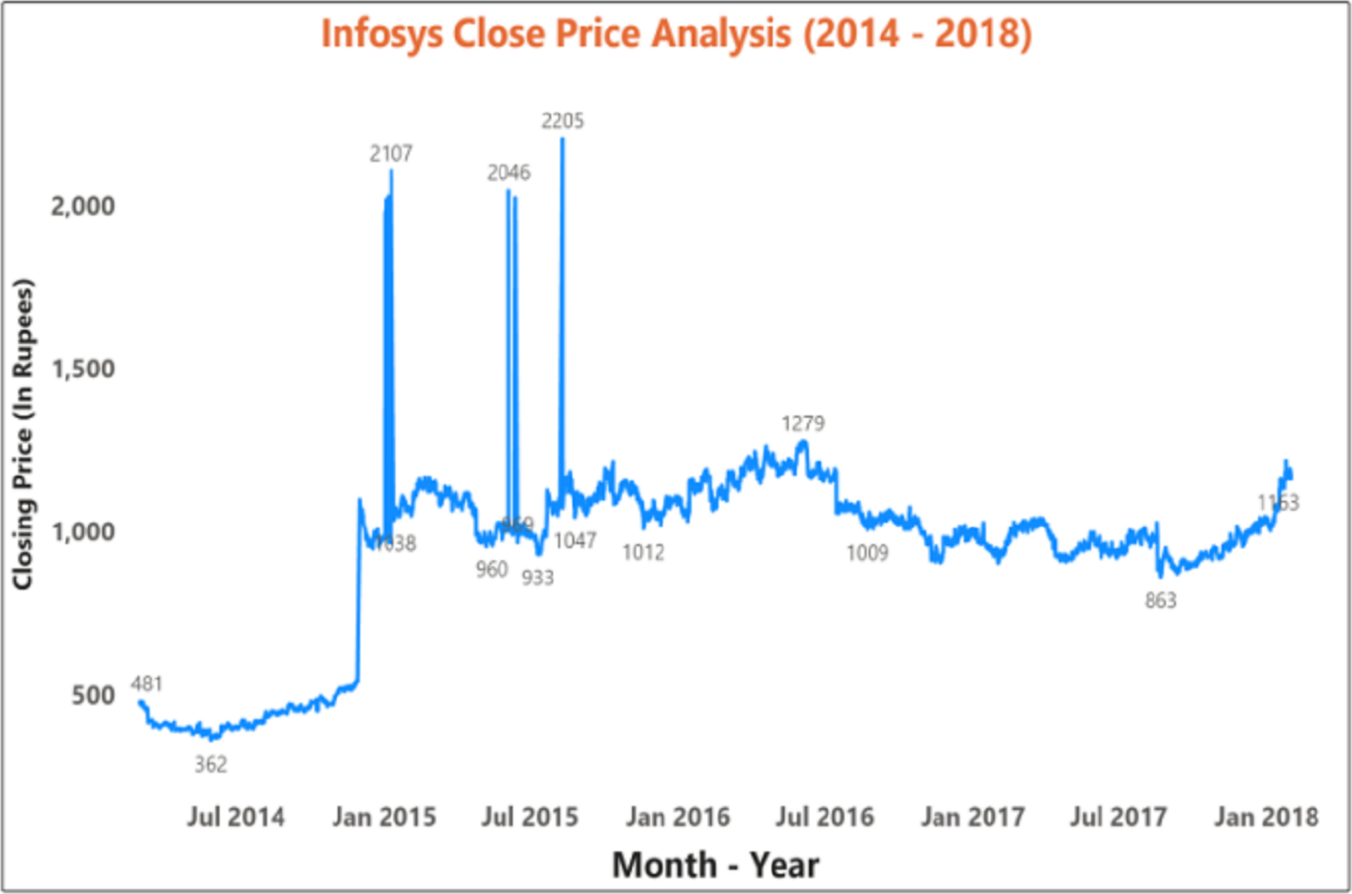
Time series data analysis of the closing price of Infosys company (2014–2018 ).

**Figure 12:**
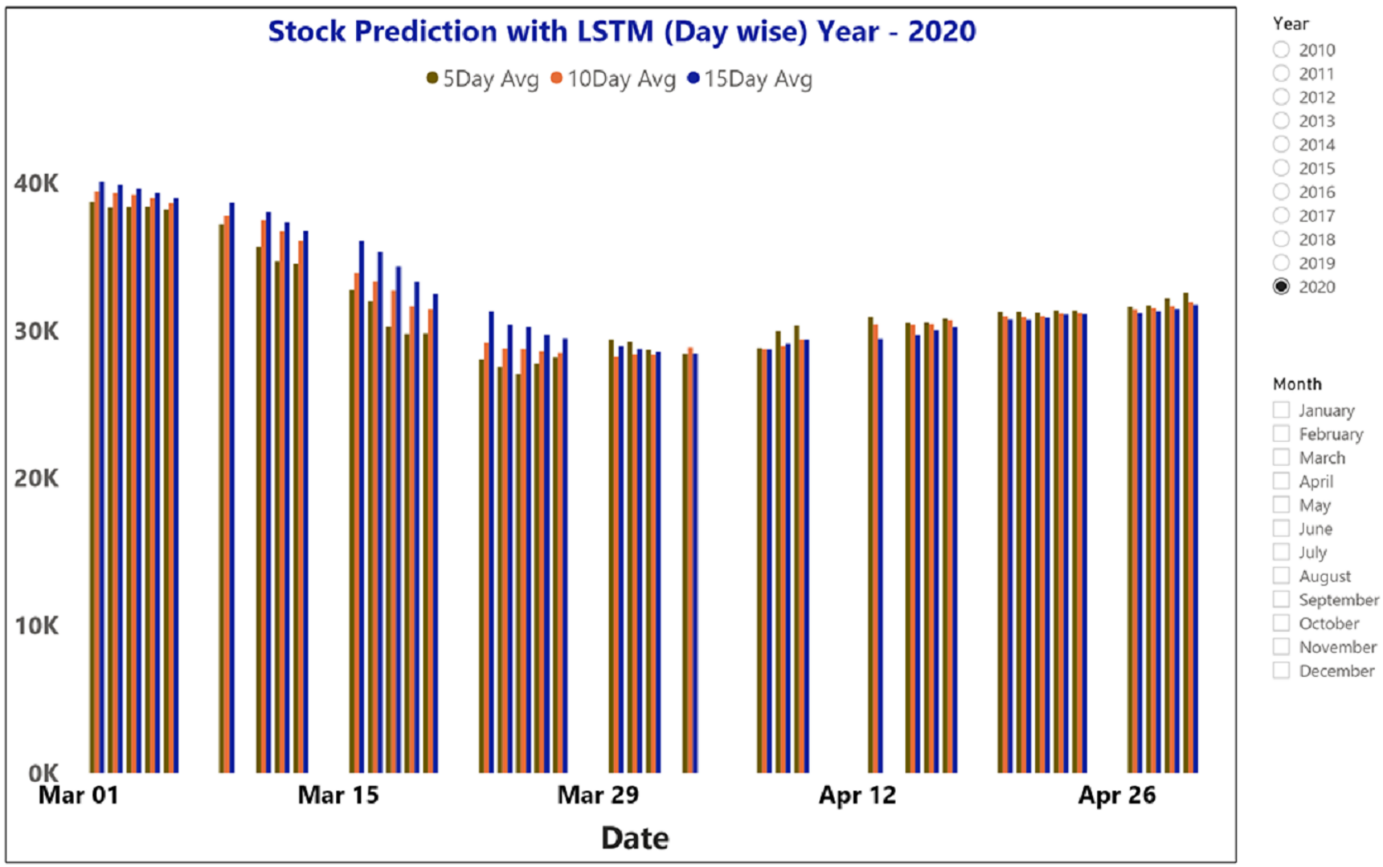
The moving average metric prediction representations for BSE sensex (March and April-2020).

**Figure 13:**
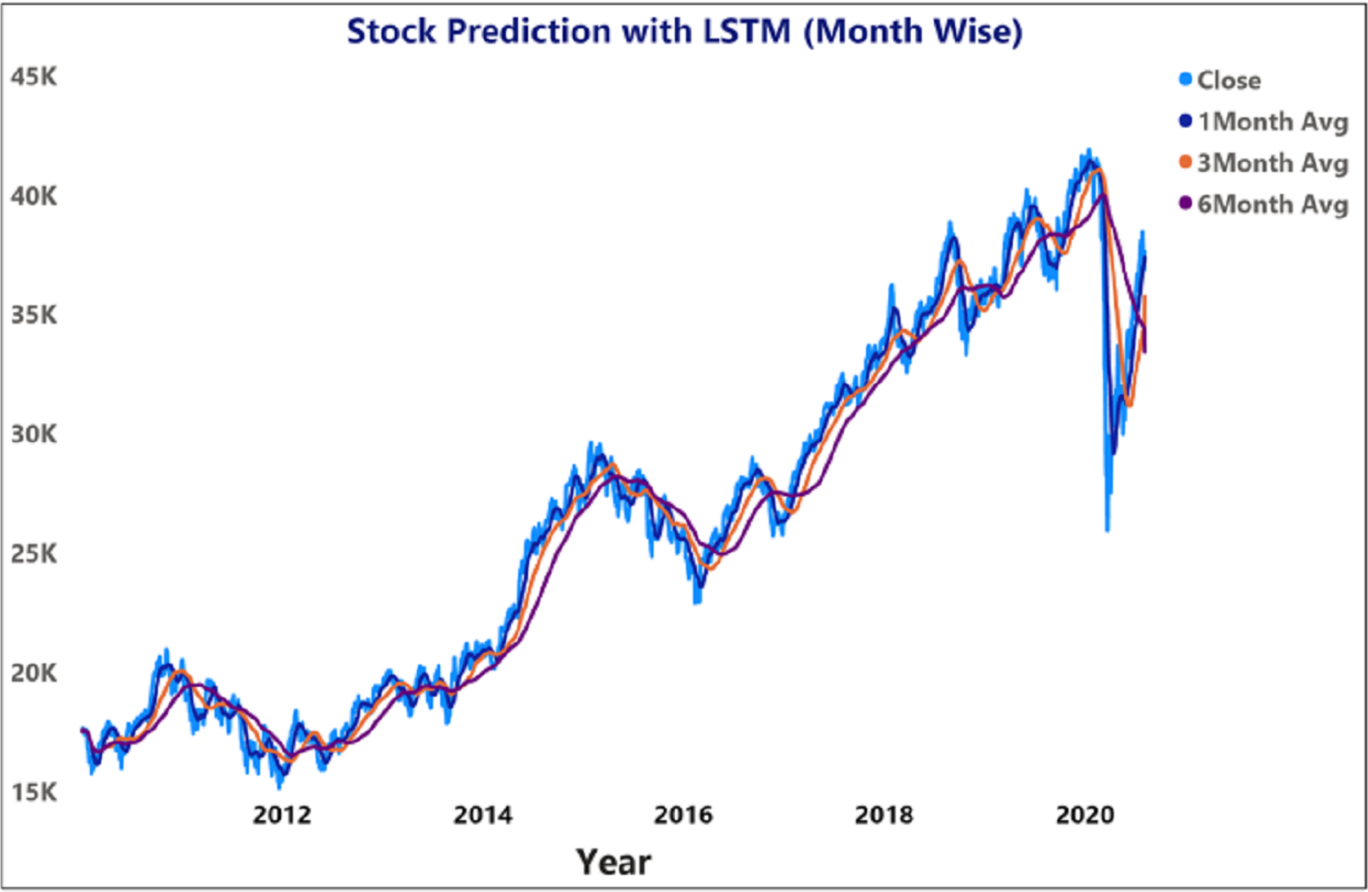
Prediction of month wise moving average of stock price using LSTM for BSE sensex.

**Figure 14:**
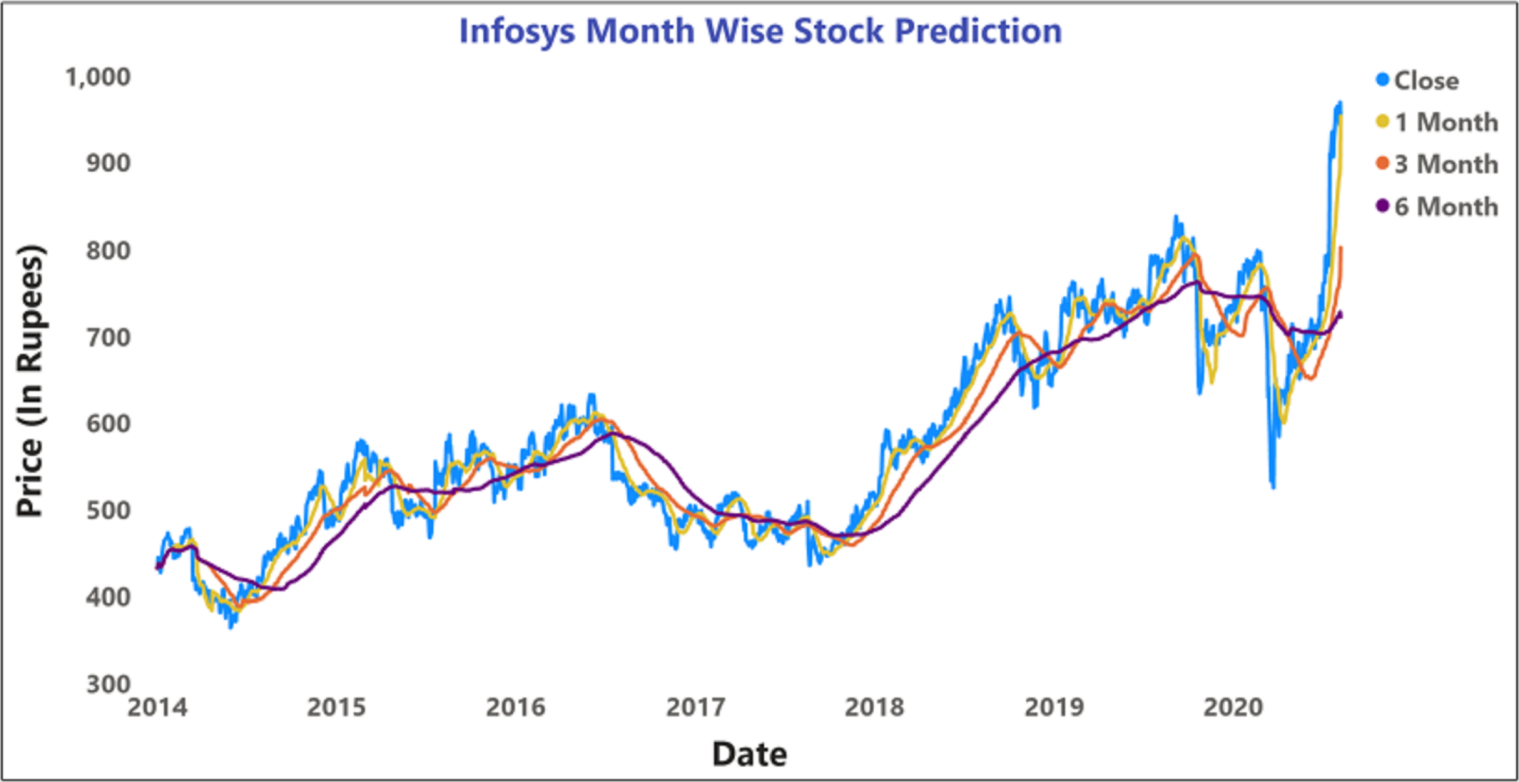
Prediction of month wise moving average of INFOSYS stock data.

Figure 11 depicts the time-series data analysis of the Infosys Company's closing price data (2014–2018). Figures 12–14 represent the accuracy prediction of numerical data concerning its sentiments. Our results indicate a strong relation between finance news data and SM prices using LSTM methodology. The moving average metric’s day-wise analysis was considered for 5, 10, and 15 days in our experiments. Furthermore, the moving average’s month-wise analysis was considered for 1, 3, and 6 months. The investigation results also forecasted the online news articles compared to the historical data of SM movements. Our primary focus was to provide a better base for prediction and forecasting to the used classification approaches such as Naïve Bayes Technique, Linear Regression, Maximum Entropy, Decision Tree, Linear SVC classifier, and LSTM.

The moving average prediction analysis was represented using a color-coded scheme: (i) 5-days (red color), (ii) 10-days (orange), and (iii) 15-days (blue). The moving average was estimated from March 2020 to April 2020.

Figures 12 and 14 represent the month-wise average calculation of the Infosys Company’s stock data predicted by LSTM from 2014 to 2020. The results of our study may assist the SM stakeholders in buying, selling, and holding individual stocks. Furthermore, it can be observed that in the case of day-wise predictions of 5, 10, and 15 days based on financial news data, the predominant sentiment correlated to the average opening and closing stock prices. The average SM movement evaluation was conducted using average Sentiment, polarity values, and the final and initial stock price values. Our study recorded gradual improvements in the day-wise SM forecasted data of 5, 10, and 15 days. In the case of 10 days, a slightly reduced performance was recorded compared to 5 days; similar results have been obtained in SM monthly data from 1, 3, and 6 months. All presented results had also considered SA opinions. Table 6 shows the accuracy metrics of various classification approaches concerning its sentiment polarity and indicates that LSTM obtained the maximum accuracy of around 92.45% using social media and financial news data.

**Table 6:** Accuracy of the given classification approaches.

ML classification techniques	Accuracy (%)
Naïve Bayes technique	86.72
Linear regression	86.75
Maximum entropy	88.93
Decision tree	81.43
Linear SVC classifier	89.46
LSTM	92.45

Linear SVC Cl obtained the second-highest accuracy classifier. The Naïve Bayes, Linear Regression, and Maximum Entropy methodology remained around 86.72%, 86.75%, and 88.93%.

## Conclusions and future scope

Assessing future stock trends is a significant task since stock movements depend on the number of factors involved. Researchers have anticipated that news articles and the share value are interrelated and that the news might correlate to fluctuations in shares. We suggested and applied the fundamental approaches for stock price prediction and focused on the SA model using different machine learning classification techniques and an in-depth learning approach using the news/social media data with historical and past stock data. We also analyzed the association between news data with stock trend values during a specific period. Polarity detection can help determine whether news sentiment can be identified as positive and negative. A positive news effect is likely to reflect that the share market values are high, and if the news is negative, then the impact of the trend is low. We used the ML classifier and deep learning technique with the stock price and news data set. The experiment shows that by using Sentiment with the historical stock price, we can get the stock’s accuracy so consumers can sell and buy their stock with stock movement. The are future opportunities for research in this area. Updated data also plays a vital role in forecasting the SM. The share markets are highly unpredictable and are typically impacted by the events in a specific country, and news may serve as a useful information source. Deep learning models are widely used in the SM prediction. There are several different layers involved in the construction of the deep learning models. This process takes time, which makes a deep learning system slow and time-consuming. Methods should be used to identify the correct sentiment and accuracy that can be achieved to make the process faster and more accurate.

Our research provided the following:

- Suggested a combination of economic news and social network analysis to forecast SM developments.
- Implemented a collection of features from final data sets to improve the performance of predictions.
- Implemented capital markets prices that are difficult to forecasting.
- Recommended more impact on the SMs from social networking sites and financial forecasts.
- Implemented an in-depth learning approach to SM analysis.

## Supplemental Information

10.7717/peerj-cs.476/supp-1 Supplemental Information 1 Data Set of Infosys and BSE.

10.7717/peerj-cs.476/supp-2 Supplemental Information 2 Sentiment Analysis Code.

## Additional Information and Declarations

### Data Availability

The following information was supplied regarding data availability: The data used in the conducted experiments and the code are available in the Supplemental Files.
